# Recapitulation of methotrexate hepatotoxicity with induced pluripotent stem cell-derived hepatocytes from patients with rheumatoid arthritis

**DOI:** 10.1186/s13287-018-1100-1

**Published:** 2018-12-29

**Authors:** Juryun Kim, Yena Kim, Jinhyeok Choi, Hyerin Jung, Kijun Lee, Jaewoo Kang, Narae Park, Yeri Alice Rim, Yoojun Nam, Ji Hyeon Ju

**Affiliations:** 1 CiSTEM laboratory, Convergent Research Consortium for Immunologic Disease, Seoul St. Mary’s Hospital, College of Medicine, The Catholic University of Korea, Seoul, 137-701 Republic of Korea; 2 Division of Rheumatology, Department of Internal Medicine, Seoul St. Mary’s Hospital, Institute of Medical Science, College of Medicine, The Catholic University of Korea, #505, Banpo-Dong, Seocho-Gu, Seoul, 137-701 Republic of Korea; 3 Department of Biomedicine & Health Sciences, Seoul St. Mary’s Hospital, College of Medicine, The Catholic University of Korea, Seoul, 137-701 Republic of Korea

**Keywords:** Induced pluripotent stem cells, Hepatocyte, Hepatocyte spheroid, Methotrexate, Drug-induced hepatotoxicity, Rheumatoid arthritis

## Abstract

**Background:**

Methotrexate (MTX) is widely used for the treatment of rheumatoid arthritis (RA). The drug is cost-effective, but sometimes causes hepatotoxicity, requiring a physician’s attention. In this study, we simulated hepatotoxicity by treating hepatocytes derived from RA patient–derived induced pluripotent stem cells (RA-iPSCs) with MTX.

**Methods:**

RA-iPSCs and healthy control iPSCs (HC-iPSCs) were established successfully. RA-iPSCs were differentiated into hepatocytes in two-dimensional (2D) monolayers and three-dimensional (3D) hepatocyte spheroid cultures; this process required growth factors such as BMP4, bFGF, HGF, and OSM. Immunofluorescence staining and flow cytometry were performed to confirm that the mature hepatocytes expressed cytokeratin 18, anti–alpha-1 antitrypsin, and albumin. MTX toxicity was evaluated via monitoring of cell viability, alanine aminotransferase, and mitochondrial status after MTX treatment in 2D and 3D cultures.

**Results:**

RA-iPSCs generated from three RA patients suffering from MTX-induced hepatotoxicity differentiated into the endoderm lineage, hepatoblasts, and hepatocytes. In 2D culture, RA-iPSC-derived hepatocytes were more sensitive to MTX than healthy controls. A 3D culture system using hepatocyte spheroids also successfully recapitulated MTX-induced hepatotoxicity. The 3D culture system had several advantages, including longer culture periods under more complex conditions.

**Conclusions:**

A patient-derived iPSC platform could recapitulate MTX toxicity. Simulation of drug toxicity in vitro may help clinicians choose safer drugs or less toxic doses.

**Electronic supplementary material:**

The online version of this article (10.1186/s13287-018-1100-1) contains supplementary material, which is available to authorized users.

## Background

Rheumatoid arthritis (RA) is a chronic disease that causes inflammation and destruction of bone and is associated with signs of systemic immune imbalance. To treat RA, drugs such as nonsteroidal anti-inflammatory drugs (NSAIDs), disease-modifying antirheumatic drugs (DMARDs), and biological reagents are prescribed according to the severity of pathology [1, 2]. Among these medications, methotrexate (MTX) is the gold standard for treatment of RA and is commonly prescribed to patients.

MTX, a folic acid antagonist derived from aminopterin, inhibits the enzyme dihydrofolate reductase and has anti-inflammatory activity [3]. However, a subset of RA patients taking this medicine for long periods exhibit drug-induced liver injury, resulting in abnormally elevated levels of aspartate aminotransferase (AST) and alanine aminotransferase (ALT). Consequently, even though MTX is cost-effective, physicians must restrict prescription of this drug due to its hepatotoxicity [4, 5].

Primary hepatocytes derived from patient liver tissue have been used to predict and measure hepatotoxicity. However, because primary hepatocytes cannot proliferate and have a short lifespan in culture, they are of limited utility for toxicology. For many years, immortal liver cancer cell lines such as HepG2 and HepaRG have been used to estimate hepatotoxicity, but their sensitivity is low, and these cell lines fail to reflect the patient’s characteristics [6, 7].

Human-induced pluripotent stem cells (iPSCs), which are induced by viruses expressing the Yamanaka factors (OCT4, Sox2, Klf4, c-Myc), can be generated from multiple types of somatic cells, including fibroblasts and peripheral blood mononuclear cells (PBMCs) [8]. Their ability to differentiate into all three germ layers enables iPSCs to produce hepatocyte-like cells, via the endodermal state, under the influence of diverse growth factors. Hepatocytes induced from iPSCs have liver function comparable to that of immortal cell lines, express albumin, and the enzyme A1AT and are suitable for use as in vitro disease models [9–12]. Moreover, the self-renewal properties of iPSCs overcome the limitations on primary hepatocyte proliferation. To date, however, no study has sought to verify hepatotoxicity in hepatocyte-like cells using iPSCs derived from RA patients’ somatic cells.

In recent work, iPSCs have been used to study hepatotoxicity [13–15]. In several studies, iPSCs were differentiated into endoderm, hepatoblasts, immature hepatocytes, and mature hepatocytes using growth factors such as Activin A, basic fibroblast growth factor (bFGF), bone morphogenetic protein 4 (BMP4), hepatocyte growth factor (HGF), and Oncostatin M (OSM) [16–19]. In addition, hepatocyte-like cells have been derived from iPSCs using small molecules such as CHIR99021 [20], and recent studies have used small molecules such as FH1 to promote maturation of differentiated hepatocyte-like cells [21]. All of these studies were conducted using two-dimensional (2D) monolayer culture and have overcome the problem of providing a cell source, which as noted above is an issue when using primary hepatocytes. However, the induced cells live only 6–10 days after the completion of differentiation, making it difficult to measure long-term toxicity or drug efficacy over long periods of time. To overcome this problem, a three-dimensional (3D) culture system similar to the in vivo environment has been developed with the goal of increasing the lifespan of primary hepatocytes (or iPSC-derived hepatocytes), as well as boosting albumin secretion and other hepatocyte functions [22, 23].

In this study, we sought to investigate the toxicity of MTX over the short term in 2D monolayer culture and furthermore to determine the toxicity of MTX over the long term by differentiating iPSC-derived hepatocytes in spheroid culture. To this end, we generated iPSCs from somatic cells of RA patients with MTX-induced hepatotoxicity or healthy controls, and then differentiated these iPSCs into hepatocyte-like cells and 3D hepatocyte spheroids using growth factors. Using the resultant hepatocyte-like cells and spheroids, we attempted to simulate MTX hepatotoxicity.

## Methods

### Differentiation of iPSCs

To generate iPSCs, PBMCs and skin cells were obtained from RA patients with hepatotoxicity caused by MTX treatment, as well as from healthy control subjects. iPSCs were generated as previously described [24]. Briefly, PBMCs obtained from each group were cultured for 4 days in StemSpan medium (STEMCELL Technologies, Vancouver, Canada) to expand CD34-positive cells. Expanded PBMCs or skin cells were transfected with the CytoTune-iPS Sendai Reprogramming Kit (Life Technologies, Carlsbad, CA, USA) including the Yamanaka factors (Oct4, Sox2, KLF4, and c-Myc). PBMCs were induced to form iPSCs by centrifugation; the resultant attached cells were expanded and purified by colony picking.

### Blood samples, skin samples, and ethics statement

The Institutional Review Board (IRB) of the Catholic University of Korea, Seoul St. Mary’s Hospital, approved this study (IRB Number: KC13TISI0775).

### Differentiation of hepatocyte-like cells

Human iPSCs were subcultured in dishes coated with vitronectin at 37 °C in an incubator containing 10% CO_2_. Fresh Essential 8 (E8) medium, replaced once per day, was used as the culture medium. iPSCs were split using trypsin-EDTA (TE) at 70% confluence, and 10 μM rho-associated kinase (ROCK) inhibitor was added to the newly passaged cells. To differentiate into endoderm, STEMdiff definitive endoderm basal medium (STEMCELL Technologies) was used as the culture medium. Briefly, on day 1, STEMdiff definitive endoderm basal medium containing supplements A and B was added to the plate containing packed iPSCs. On days 2–4, the medium was replaced with medium prepared by diluting STEMdiff definitive endoderm basal medium with only supplement B. On days 5–7, the plate was washed with RPMI 1640 medium, and the medium was replaced with RPMI/B27 medium supplemented with 10 ng/mL bFGF and 20 ng/mL BMP4. On day 8, the medium was replaced with hepatocyte basal medium (HBM) (Lonza, Basel, Switzerland) containing 50 ng/mL HGF (Peprotech, NJ, USA) and 30 ng/mL OSM (R&D Systems, Minneapolis, MN, USA). To re-plate hepatoblasts into proper microplates, the cells were dissociated with TE and seeded into 96-well plates or the indicated culture vessel. For passage, hepatoblast medium was removed from culture plates, the plates were washed with sterile phosphate-buffered saline (PBS), and the cells were dissociated with TE. Hepatoblasts were collected with hepatoblast basal medium and pelleted by centrifugation (1100 rpm, 27 °C, 2 min). For 96-well plates, 3.3 × 10^4^ hepatoblasts were seeded in growth-factor-reduced Matrigel (BD Biosciences, San Jose, CA, USA)-coated wells in hepatocyte medium supplemented with 50 ng/mL HGF (Peprotech), 30 ng/mL OSM (R&D), and ROCK inhibitor. From day 10 until day 26, new hepatocyte medium was replaced with 50 ng/mL HGF and 30 ng/mL OSM every other day.

### 3D hepatocyte spheroid differentiation

A 96-well round-bottomed plate was coated for 5 min with anti-adherent rinsing solution (STEMCELL Technologies) and washed once with PBS. To prepare culture medium for generation of spheroids, HBM was supplemented with 50 ng/mL HGF, 30 ng/mL OSM, and 1:100 (*v*/*v*) BD Matrigel™ Basement Membrane Matrix (BD Biosciences, San Jose, CA, USA). On day 8 of differentiation, hepatoblasts differentiated from iPSCs were dissociated with TE. Five thousand hepatoblast cells in 100 μL of medium, prepared as described above, were seeded in each well of the pre-coated 96-well round-bottomed plate, which was then centrifuged at 300*g* for 3 min. Subsequently, the medium was replaced every other day with HBM containing 50 ng/mL HGF and 30 ng/mL OSM.

### Real-time PCR

RNA was extracted from iPSCs using TRIzol (Life Technologies), and cDNA was synthesized using RevertAid™ First Strand cDNA Synthesis Kit (Thermo Fisher Scientific, Waltham, MA, USA). Real-time PCR was performed using SYBR Green real-time PCR master mix (Roche, Basel, Switzerland) and RT PCR was performed using i-Taq™ DNA Polymerase (iNtRON BIOTECHNOLOGY, Seongnam, South Korea). Primer sequences are shown in Table 1.

**Table 1:** Sequences of primers used for PCR

Target name	Direction	Primer sequence (5′–3′)	Size (bp)
OCT3/4	Forward	ACCCCTGGTGCCGTGAA	190
	Reverse	GGCTGAATACCTTCCCAAATA	
SOX2	Forward	CAGCGCATGGACAGTTAC	321
	Reverse	GGAGTGGGAGGAAGAGGT	
NANOG	Forward	AAAGGCAAACAACCCACT	270
	Reverse	GCTATTCTTCGGCCAGTT	
DPPA5	Forward	CGGCTGCTGAAAGCCATTTT	215
	Reverse	AGTTTGAGCATCCCTCGCTC	
LIN28	Forward	GTTCGGCTTCCTGTCCAT	122
	Reverse	CTGCCTCACCCTCCTTCA	
ALB	Forward	TTGGCACAATGAAGTGGGTA	161
	Reverse	AAAGGCAATCAACACCAAGG	
CYP1A2	Forward	ATGGCATTGTCCCAGTCTGTT	135
	Reverse	TGGCTCTGGTGGACTTTTCAG	
CYP2D6	Forward	GTGTCCAACAGGAGATCGACG	101
	Reverse	CACCTCATGAATCACGGCAGT	
CYP2E1	Forward	GTTCTTTGCGGGGACAGAGA	202
	Reverse	GAGGGTGATGAACCGCTGAA	
CYP3A4	Forward	TGTGCCTGAGAACACCAGAG	226
	Reverse	GTGGTGGAAATAGTCCCGTG	
CYP3A7	Forward	GAAACACAGATCCCCCTGAA	105
	Reverse	TCAGGCTCCACTTACGGTCT	
UGT1A1	Forward	CAGCAGAGGGGACATGAAAT	174
	Reverse	ACGCTGCAGGAAAGAATCAT	
UGT2B15	Forward	GTGTTGGGAATATTATGACTACAGTAAC	157
	Reverse	GGGTATGTTAAATAGTTCAGCCAGT	
OATP1B1	Forward	GAGCAACAGTATGGTCAGCCT	135
	Reverse	GGCAATTCCAACGGTGTTCA	
OATP1B3	Forward	GTCCAGTCATTGGCTTTGCA	111
	Reverse	CAACCCAACGAGAGTCCTTAGG	
NTCP	Forward	GGGACATGAACCTCAGCATT	199
	Reverse	CGTTTGGATTTGAGGACGAT	
MRP2	Forward	AGCGTCCTCTGACACTCG	206
	Reverse	GGCATCTTGGCTTTGACT	
MDR1	Forward	CTAATGCCGAACACATTGGA	237
	Reverse	CAGTCGCTTTATTTCTTTGCC	
AHR	Forward	CAAATCCTTCCAAGCGGCATA	123
	Reverse	CGCTGAGCCTAAGAACTGAAAG	
FXR	Forward	CAGGATTTCAGACTTTGGACCAT	63
	Reverse	CTTCAACCGCAGACCCTTTC	
GR	Forward	ATAGCTCTGTTCCAGACTCAACT	111
	Reverse	TCCTGAAACCTGGTATTGCCT	
PPARα	Forward	AGAGATTTCGCAATCCATCGG	62
	Reverse	ACTGGTATTCCGTAAAGCCAAAG	
RXRA	Forward	ATGGACACCAAACATTTCCTGC	211
	Reverse	GGGAGCTGATGACCGAGAAAG	
SHP	Forward	CCCCAAGGAATATGCCTGCC	233
	Reverse	TAGGGCGAAAGAAGAGGTCCC	
ADORA1	Forward	GTCCTCATCCTCACCCAGAG	189
	Reverse	CAGATTGTTCCAGCCAAACA	
ADORA2A	Forward	CGAGGGCTAAGGGCATCATTG	98
	Reverse	CTCCTTTGGCTGACCGCAGTT	
ADORA2B	Forward	CTCTTCCTCGCCTGCTTCGTG	107
	Reverse	TTATACCTGAGCGGGACACAG	
ADORA3	Forward	TACATCATTCGGAACAAACTC	80
	Reverse	GTCTTGAACTCCCGTCCATAA	
CD39	Forward	ACAGGCGTGGTGCATCAAGTAGAA	279
	Reverse	CCTGGCACCCTGGAAGTCAAAG	
CD73	Forward	CAGTACCAGGGCACTATCTG	194
	Reverse	AGTGGCCCCTTTGCTTTAAT	
GAPDH	Forward	GAATGGGCAGCCGTTAGGAA	414
	Reverse	GACTCCACGACGTACTCAGC	

### Quantitative analysis of gene expression for drug metabolism

RNA was extracted from HepG2 cells and iPSCs using TRIzol when cells were 60~70% confluent after seeding. In the case of iPSC-derived hepatocyte, RNA was extracted using TRIzol after completion of differentiation. cDNA was synthesized and real-time PCR was performed using primers of phases I, II, and III and nuclear receptor. Real-time PCR was conducted using a LightCycler® 480 Instrument II (Roche, Basel, Switzerland). Mean cycle threshold values of target gene expression was normalized with the value of the housekeeping gene, glyceraldehyde 3-phosphate dehydrogenase (*GAPDH*). All experiments were performed in triplicate. Primer sequences are shown in Table 1.

### Immunofluorescence staining

On day 8, hepatoblasts were seeded into 12-well plates containing cover glasses pre-coated with Matrigel. Cells were differentiated into hepatocyte-like cells as described in the differentiation protocol above. After two washes with 500 μL of PBS, the cells were incubated with 4% paraformaldehyde for 30 min, washed two more times with PBS, incubated with 50 mM NH_4_Cl solution for 10 min, washed again twice with PBS, incubated with 500 μL of 0.1% Triton X-100 for 10 min, incubated with 500 μL of 2% PBA for 30 min, incubated at room temperature for 2 h with primary antibodies diluted in fresh 2% PBA blocking buffer (anti-albumin, 1:200; anti-A1AT, 1:200; anti-AFP, 1:1000; anti-CK18, 1:500; anti-CYP3A4, 1:100), washed two times with 500 μL of 2% PBA, and then incubated for 1 h at room temperature with fluorescence-conjugated secondary antibody diluted in 2% PBA buffer. After washing, the cells were mounted using antifade and visualized on a fluorescence microscope.

### Periodic acid–Schiff staining

On day 8, hepatoblasts were seeded in 12-well plates pre-coated with Matrigel. Cells were cultured until day 26 in HBM as described in the differentiation protocol above. After two washes with 500 μL of PBS, the cells were incubated with 4% paraformaldehyde for 30 min, washed with PBS, incubated for 10 min with 0.1% Triton X-100, acidified for 5 min with 1% periodic acid (Sigma-Aldrich, St. Louis, MO, USA), washed three times with distilled water, and treated with Schiff’s reagent for 20 min. After Schiff’s reagent was aspirated, the cells were washed in distilled water for 5 min. After replacement of the wash solution with fresh distilled water, staining was visualized under a light microscope.

### CCK-8 assay

Human iPSCs were induced to form hepatocytes as described above. On day 8, the cells were re-seeded into 96-well microplates (3.3 × 10^4^ cells/well). On day 21 of hepatocyte differentiation, MTX was administered at a dose of 0 nM, 1 nM, 10 nM, 100 nM, 1 μM, 10 μM, or 100 μM, and the cells were cultured for 6 days with a medium change every other day. The cells were then treated with 10 μL of CCK-8 solution per well and incubated for 30 min~4 h at 37 °C in an incubator, and absorbance was measured at 450 nm.

### Flow cytometry

Hepatocyte-like cells derived from iPSCs were dissociated using Accumax (Innovative Cell Technologies, San Diego, CA, USA). Cells were washed twice with PBS containing 2% FBS, permeabilized for 30 min using Flow Cytometry Fixation and Permeabilization Buffer (Thermo Fisher Scientific, MA, USA), washed with wash buffer, stained with allophycocyanin (APC)-conjugated human albumin antibody (R&D Systems) and Oct4 antibody (Abcam, Cambridge, UK) for 1 h each, and then washed with wash buffer. Analysis was performed on a BD LSRFortessa cell analyzer (BD Biosciences), and data were analyzed using the FlowJo V10 Single Cell Analysis Software (TreeStar Inc., OR, USA).

### ALT activity assay

Starting on day 21 of hepatocyte differentiation, MTX was administered at a dose of 0 nM, 1 nM, 10 nM, 100 nM, 1 μM, 10 μM, or 100 μM once every 2 days for 6 days. Culture supernatants were collected, and ALT activity was detected manually using the Alanine Aminotransferase activity assay (Sigma-Aldrich). Briefly, culture supernatant in each well of a 96-well plate was mixed with a master reaction mix containing ALT assay buffer, fluorescent peroxidase substrate, ALT enzyme mix, and sub-substrate. Initial fluorescence intensity (*λ*_ex_ = 535; *λ*_em_ = 587 nm) was measured, and the sample was incubated at 37 °C in the dark and assayed every 5 min until the measured value of the most active sample was greater than the highest standard value.

### 3D spheroid cell viability assay (ATP assay)

Starting on day 21 of hepatocyte differentiation, MTX was administered at a dose of 0 nM, 1 nM, 10 nM, 100 nM, 1 μM, 10 μM, or 100 μM once every 2 days for 14 days. The CellTiter-Glo 3D cell viability assay reagent (Promega, Madison, WI, USA) was dissolved the day before and left at room temperature for 30 min before use. Spheroids in 100 μL of culture medium were added to 100 μL of the CellTiter-Glo 3D cell viability assay reagent and mixed vigorously for 30 min to lyse the cells. Absorbance was measured using a Luminometer (Spectra Max L, USA).

### Live/dead cell staining

Starting on day 21 of hepatocyte differentiation, MTX was administered at a dose of 0 nM, 1 nM, 10 nM, 100 nM, 1 μM, 10 μM, or 100 μM once every 2 days for 14 days. Ethidium homodimer and Calcein AM from the Live/DEAD Viability/Cytotoxicity Kit (Thermo Fisher Scientific) were slowly dissolved at room temperature. Ethidium homodimer was diluted 1:500 in PBS, and Calcein AM was diluted 1:2000; 200 μL of both solutions was added to hepatocyte spheroids. After incubation at 37 °C for 30 min, the cells were washed with PBS, and a z-stack fluorescence image was obtained using a confocal microscope.

### Mitochondrial staining

Starting on day 21 of hepatocyte differentiation, MTX was administered every other day for 14 days. After washing once with PBS, 10 μM Mitotracker Orange CM-H2TMROS (Thermo Fisher Scientific) and 200 nM Hoechst 33342 (Thermo Fisher Scientific) were added to the samples. After incubation for 30 min, a z-stack fluorescence image was obtained on a confocal microscope.

### Statistical analysis

The GraphPad Prism software (v. 5.01; GraphPad, San Diego, CA, USA) was used for statistical analysis. Statistical significance was assessed by Student’s *t* test and is expressed as follows: *, *p* < 0.05; **, *p* < 0.01; ***, *p* < 0.001.

## Results

### Generation of human iPSCs from a RA patient with MTX-induced hepatotoxicity

**Fig. 1:**
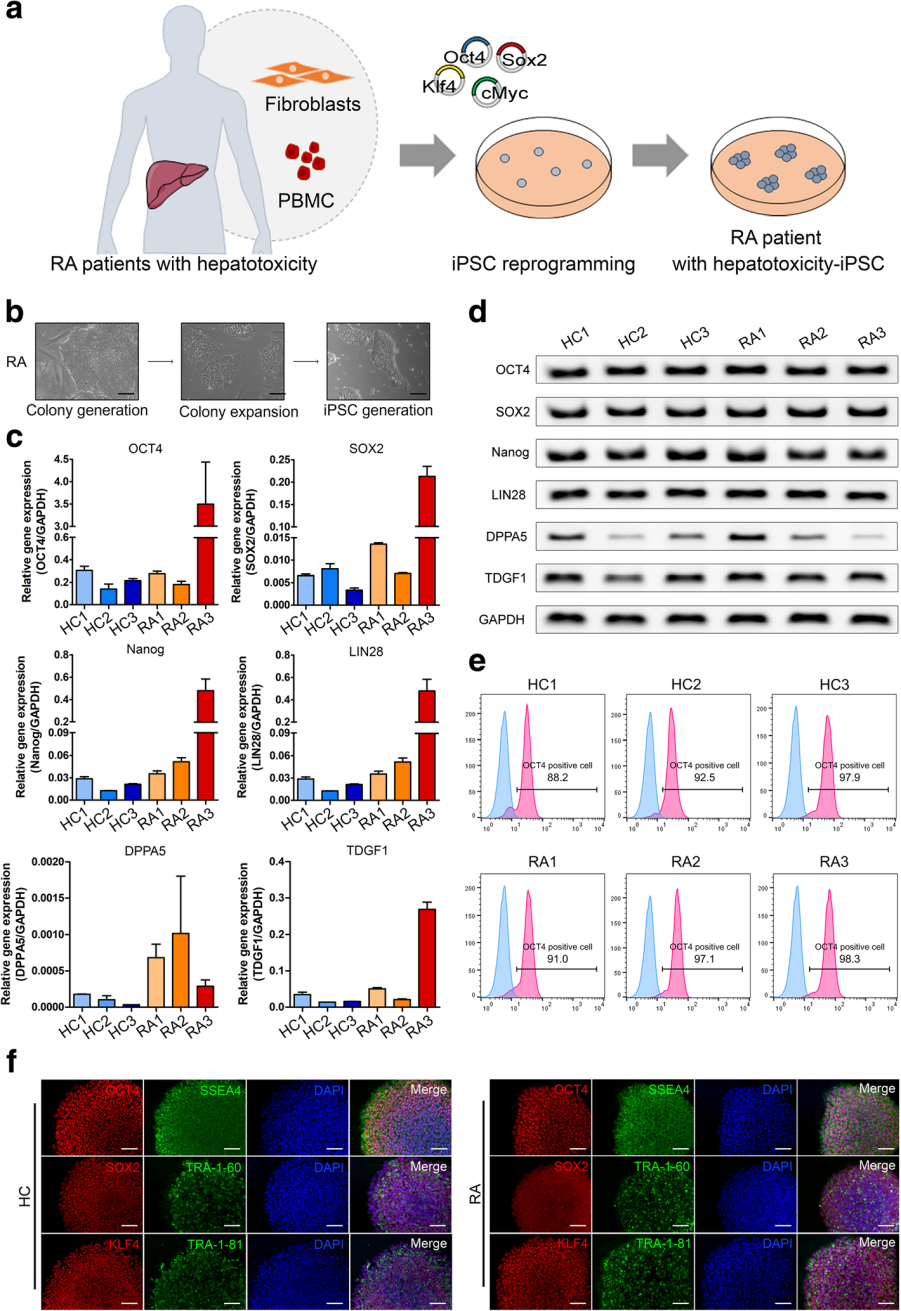
Generation of iPSCs from healthy control subjects and RA patients with MTX-induced hepatotoxicity. **a** Scheme of generation of iPSCs from healthy controls and RA patients with MTX-induced hepatotoxicity. **b** Induction of iPSCs derived from RA patients with hepatotoxicity. **c** Real-time PCR data for pluripotency gene expression in iPSCs. **d** RT-PCR data for pluripotency gene expression in iPSCs. **e** Flow cytometry data of iPSCs showing a population of OCT3/4-positive cells. **f** Immunocytochemistry images showing that pluripotent markers (SSEA4, OCT3/4, TRA-1-60, Sox 2, TRA-1-81, and Klf4) were expressed in iPSCs from healthy control (HC)- and RA patient (RA)-iPSCs. Scale bars, 200 μm

Somatic cells, such as skin fibroblasts or PBMCs, from healthy control subjects (*n* = 3) and RA patients with MTX-induced hepatotoxicity (*n* = 3) were induced to form iPSCs with Sendai virus expressing the Yamanaka factors (Oct4, Sox2, KLF4, c-Myc). The reprogramming method was based on a previous protocol using serial centrifugation [24]. Colonies were generated from somatic cells in about 18 days (Fig. 1a, b). RA patients had AST levels of 63–170 IU/L and ALT levels of 122–220 IU/L within 1 week after MTX administration (Table 2).

**Table 2:** ALT and AST levels and *MTHFR* mutation analysis in RA patients

Patient	Gender	Hepatotoxicity	MTX treatment	AST (IU/L)	ALT (IU/L)	*MTHFR* mutation C677T	*MTHFR* mutation A1298C
HC1	Male	No	No	NA	NA	Hetero (C/T)	Hetero (A/C)
HC2	Female	No	No	NA	NA	Wild (C/C)	Hetero (A/C)
HC3	Female	No	No	NA	NA	NA	NA
RA1	Female	Yes	15 mg/week	170	220	Homo (T/T)	Wild (A/A)
RA2	Male	Yes	7.5–15 mg/week	57	131	Wild (C/C)	Wild (A/A)
RA3	Male	Yes	7.5–10 mg/week	63	122	Hetero (C/T)	Hetero (A/C)

PCR analysis of gene expression revealed that the iPSCs expressed *GAPDH*, *OCT3/4*, *NANOG*, *SOX2*, *TDGF*, and *DPPA5* (Fig. 1c, d). Flow cytometry revealed that about 90% in iPSCs were positive for pluripotency marker, OCT3/4 (Fig. 1e). In addition, we confirmed the expression of pluripotency markers OCT3/4, SSEA4, TRA-1-60, SOX2, TRA-1-81, and KLF4 at the protein level by immunofluorescence (Fig. 1f, Additional file 1a, b). To determine whether the generated iPSCs were pluripotent, we subjected them to alkaline phosphatase (AP) staining. iPSCs from three healthy controls and three RA patients with hepatotoxicity stained positively for AP, indicating that they were all pluripotent and had not yet differentiated into any of the germ layers (Additional file 1c, d).

### Differentiation of hepatocytes from iPSCs in 2D monolayer culture

**Fig. 2:**
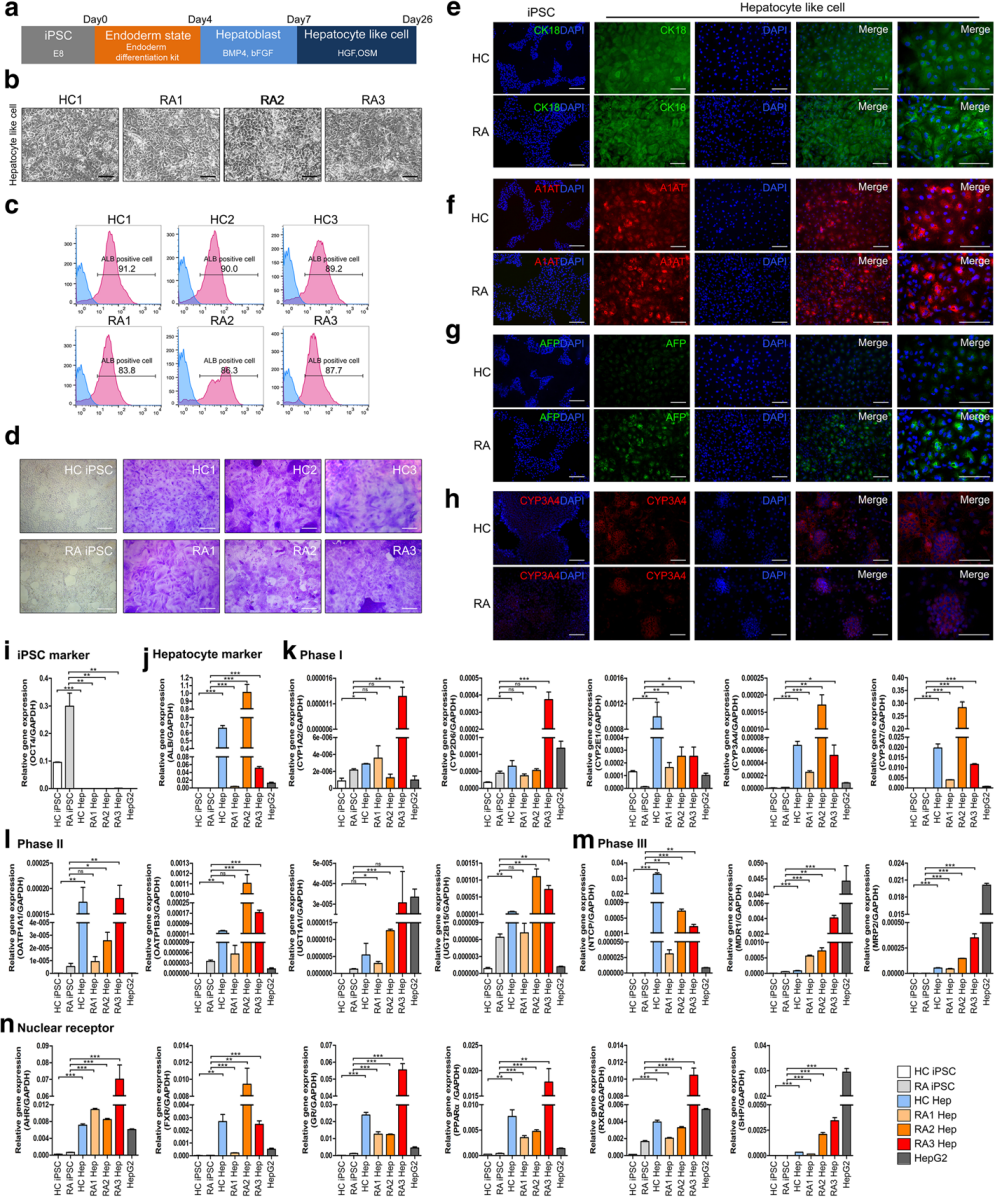
Differentiation of iPSCs derived from RA patients with MTX-induced hepatotoxicity into hepatocyte-like cells. **a** Scheme of differentiation of iPSCs from healthy control and RA patients with MTX-induced hepatotoxicity using 2D monolayer culture. **b** Microscope images showing the morphology of hepatocyte-like cells differentiated over the course of 26 days. **c** Flow cytometry data of hepatocyte-like cells showing a population of albumin-positive cells. **d** Microscope image of periodic acid staining for detection of glycogen storage in differentiated hepatocyte-like cells. **e**–**h** Immunocytochemistry of the indicated hepatocyte markers (CK18, A1AT, AFP, and CYP3A4). **i**–**n** Real-time PCR data for *OCT4*, albumin, phases I, II, and III and nuclear receptors that are related with hepatocyte function. Scale bars, 200 μm

We prepared iPSC-derived hepatocyte-like cells resembling primary hepatocytes, which are difficult to cultivate in vitro, and attempted to use these cells to simulate the hepatotoxicity resulting from MTX administration in RA patients. Human iPSCs can be differentiated into three lineages (endoderm, mesoderm, ectoderm); in particular, iPSCs can be directly differentiated into endoderm and then into hepatocytes. We used a modified protocol employing growth factors [25] in which the cells progressed from endoderm to hepatoblast to hepatocyte-like cells; all cells had differentiated after 26 days (Fig. 2a). Differentiation into the endoderm and hepatoblast states was confirmed by expression of SOX17, an endoderm marker, and HNF4α, a hepatoblast marker, as determined by immunofluorescence (Additional file 2). Hepatocyte-like cells differentiated from iPSCs exhibited cell morphology similar to that of primary hepatocytes (Fig. 2b) [26]. Flow cytometry revealed that more than 80% of hepatocyte-like cells from both healthy controls and RA patients were positive for albumin, a hepatocyte marker (Fig. 2c). Moreover, periodic acid–Schiff (PAS) staining, which indicates glycogen storage function, was positive in cells derived from both healthy controls and RA patients and there was no significant difference between HC and RA groups (Fig. 2d). Expression of the hepatocyte marker CK18 (Fig. 2e) and A1AT marker (Fig. 2f) was confirmed by immunofluorescence assay (IFA), indicating that iPSCs were well differentiated into hepatocyte-like cells in both groups. In the case of AFP, a marker of immature hepatocytes, expression was significantly lower in controls than in the RA samples, indicating that iPSC-derived hepatocyte-like cells from RA patients had a higher proportion of immature hepatocytes (Fig. 2g). In the case of CYP3A4, another marker of mature hepatocytes, expression was significantly higher in controls than in the RA samples, indicating that iPSC-derived hepatocyte-like cells from RA patients had a lower proportion of mature hepatocytes (Fig. 2h). And gene expression for drug metabolism phases I, II, and III and nuclear receptors [27] was examined in iPSC-derived hepatocyte-like cells, iPSC (negative control) and HepG2 (positive control). First, *OCT4*, a pluripotency marker expressed in iPSC, was significantly reduced in iPSC-derived hepatocyte-like cells (Fig. 2i), and albumin expressed in hepatocytes was increased in hepatocyte-like cells compared to iPSCs (Fig. 2j). In the case of *CYP3A4*, *CYP3A7*, and *CYP2E1* among Cytochrome P450 genes related to detoxicification of drug, HC iPSC and RA iPSC, which are undifferentiated cells, did not express well, but the markers were significantly increased in hepatocyte-like cells (Fig. 2k)*. UGT1A1, UGT2B15, OATP1B1, OATP1B3*, *NTCP, MRP2,* and *MDR1* genes belonging to phases II and III, drug transporter gene, also significantly increased in hepatocyte-like cells compared with iPSCs (Fig. 2l–m). The expression of *AHR, FXR, GR, PPARα, RXRA,* and *SHP* genes, the nuclear receptors that regulate the activity of CYP 450 enzyme, was also increased in hepatocyte-like cells (Fig. 2n). Together, these findings demonstrate that iPSCs could be differentiated through endoderm and hepatoblast states into hepatocyte-like cells with hepatocyte function and expression of hepatocyte markers by the 2D monolayer method.

### Recapitulation of MTX hepatotoxicity using 2D-cultured iPSC-derived hepatocytes

**Fig. 3:**
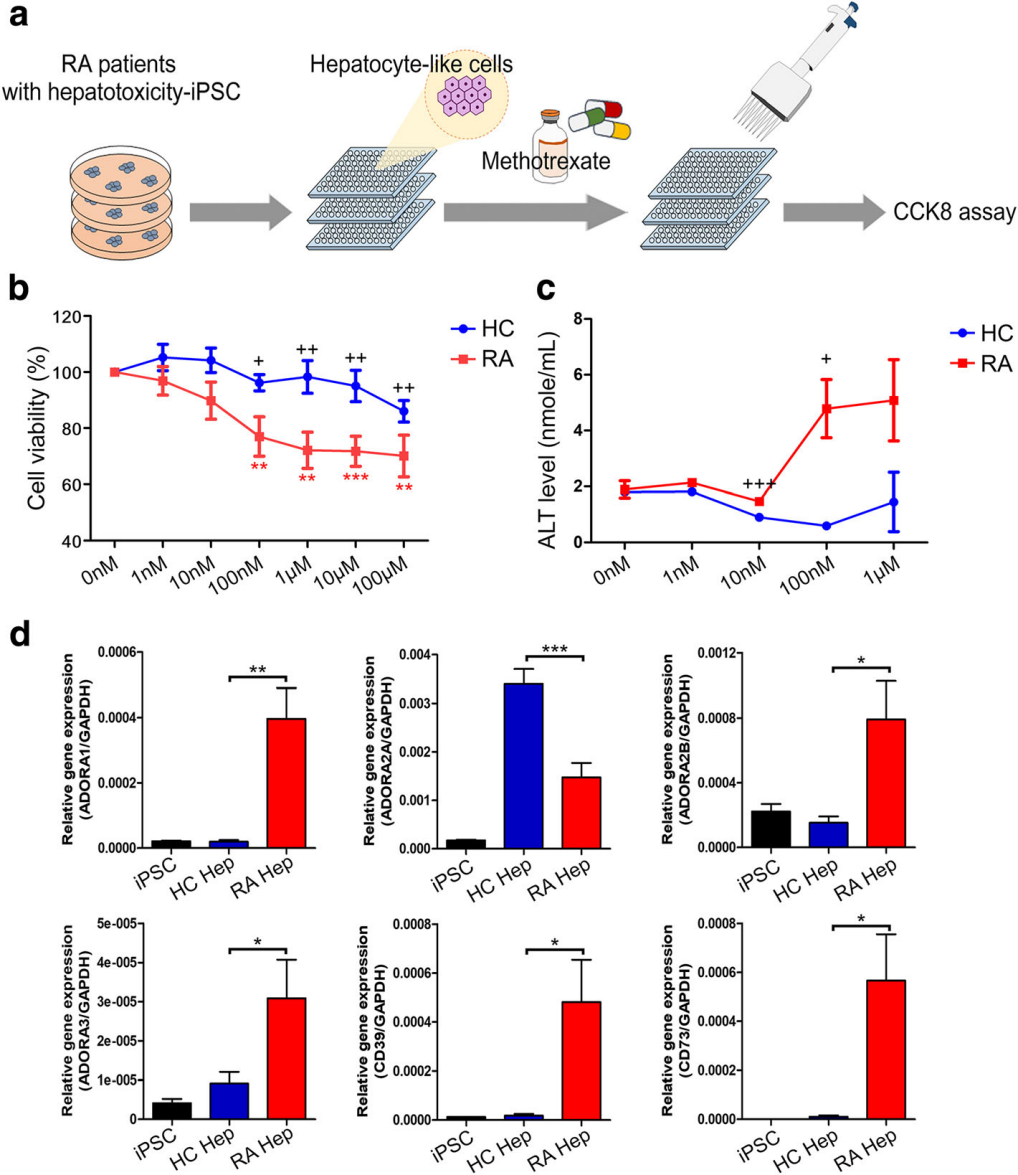
Hepatotoxicity assay of hepatocyte-like cells derived from iPSCs from RA patients with hepatotoxicity. **a** Scheme of cytotoxicity assay using hepatocyte-like cells treated with MTX for 6 days. **b** CCK-8 assay data of hepatocyte-like cells derived from iPSCs from healthy controls and RA patients with hepatotoxicity. HC: healthy control group (*n* = 3), blue line; RA: RA patient group (*n* = 3), red line. **c** ALT level of hepatocyte-like cells derived from iPSCs from healthy control and RA patient with hepatotoxicity. **d** Real-time PCR data for ADORA receptors, CD39 and CD73 gene expression in hepatocyte-like cells and iPSCs. Statistical significance is expressed as *p* value (Student’s *t* test) vs. 0 nM: *p < 0.05; **p < 0.01; ***p < 0.001. Statistical significance is also expressed as *p* value (Student’s *t* test) comparing HC and RA at each concentration: +*p* < 0.05; ++*p* < 0.01; +++*p* < 0.001

To examine MTX toxicity using hepatocyte-like cells differentiated by the 2D monolayer method, hepatoblasts were re-seeded on day 8 of differentiation in 96-well plates (*n* = 3 for healthy controls, *n* = 3 for RA patients) (Fig. 3a). Differentiation of hepatocyte-like cells was carried out in HBM with HGF and OSM until day 26. MTX was administered for 6 days, from day 21 to day 26. MTX was added at doses of 0 nM, 1 nM, 10 nM, 100 nM, 1 μM, 10 μM, or 100 μM, and hepatotoxicity was confirmed by CCK-8 assay. In RA patients, cell viability was decreased from 100% at 0 nM MTX to 60% at 100 μM. By contrast, healthy controls were resistant to MTX-induced toxicity, and maintained viability above 80% at 100 μM MTX (Fig. 3b). In cells derived from RA patients with hepatotoxicity, cell viability decreased significantly in 100 nM (*p* < 0.01 vs. 0 nM), 1 μM (*p* < 0.01), 10 μM (*p* < 0.001), and 100 μM (*p* < 0.01) MTX relative to 0 nM. ALT level in culture supernatant on day 26 was significantly higher at 100 nM in cells derived from RA patient (Fig. 3c). These results suggested that hepatocyte-like cells derived from iPSCs of RA patients with MTX-induced toxicity are more sensitive to 100 nM MTX and that iPSC-derived hepatocyte-like cells from healthy controls and RA patient are suitable for simulating MTX toxicity.

Adenosine receptors A1, A2A, A2B, A3, CD39, and CD73 are known to be associated with MTX acceptance and hepatotoxicity [28–30]. Therefore, we examined the difference in gene expression of iPSC-derived hepatocyte-like cells between healthy control and RA patient with hepatotoxicity by real-time PCR. In this study, ADORA1, ADORA2B, ADORA3, CD39, and CD73 expression were higher in RA patient group with hepatotoxicity than that of HC. In contrast, ADORA2A was expressed lower in the RA patient group with hepatotoxicity than that of HC (Fig. 3d).

### Recapitulation of MTX-induced hepatotoxicity in 3D hepatocyte spheroids

**Fig. 4:**
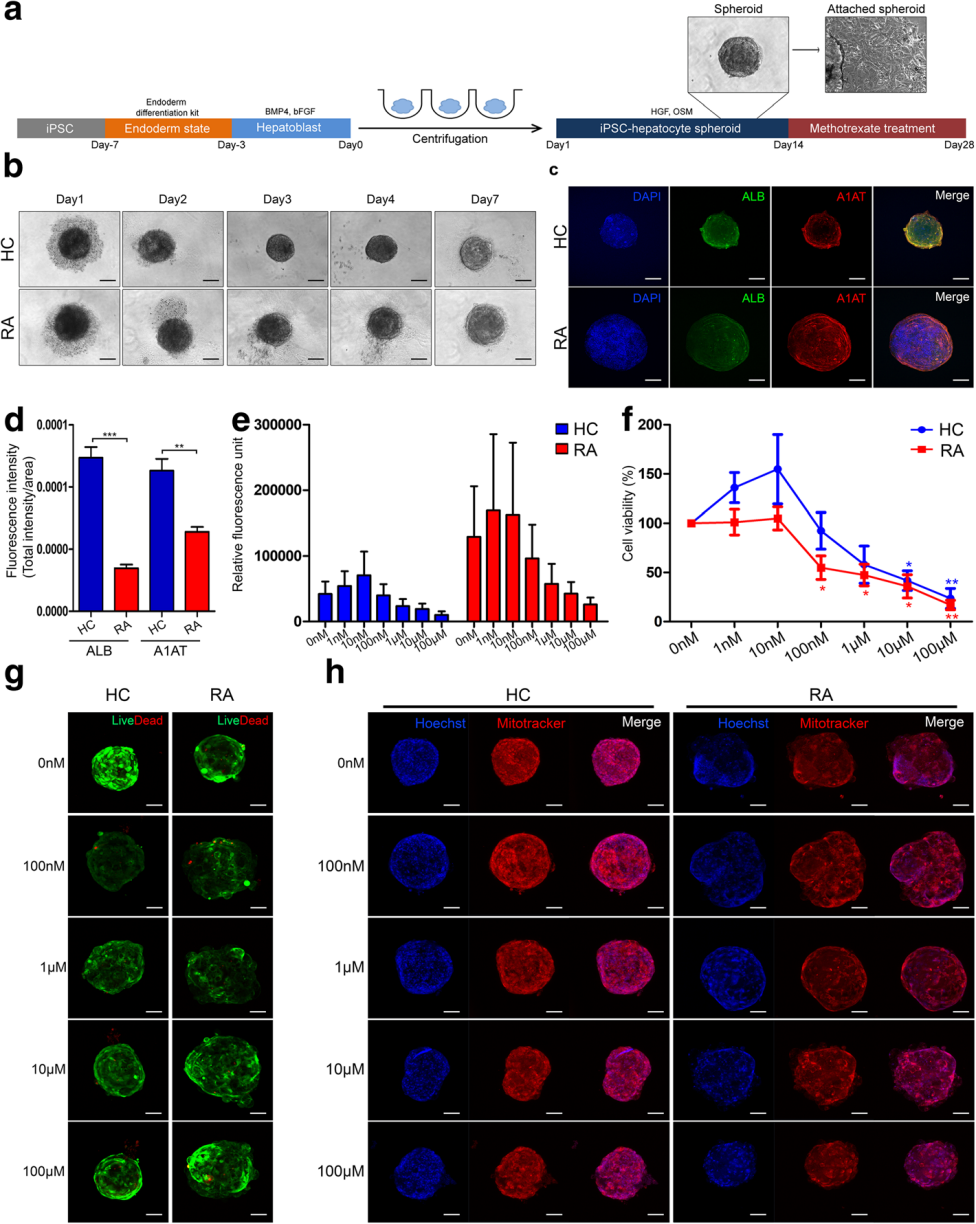
Generation and hepatotoxicity assay of hepatocyte spheroids from iPSCs derived from RA patients. **a** Scheme of generation of 3D hepatocyte spheroids and morphology of attached spheroids. **b** Morphology of hepatocyte spheroids generated from HC- and RA-iPSCs. **c** z-stack confocal microscope image of hepatocyte spheroids expressing albumin and A1AT. **d** The fluorescence quantity per area of the spheroid calculated by image J program. **e** 3D spheroid cell viability. HC: healthy control group (*n* = 3), blue bar; RA: RA patient group (*n* = 3), red bar. **f** Cell viability data relative to vehicle (MTX, 0 nM). HC: healthy control group (*n* = 3), blue line; RA: RA patient group (*n* = 3), red line. Statistical significance is expressed as *p* value (Student’s *t* test) vs. 0 nM: *p < 0.05; **p < 0.01; ***p < 0.001. **g** Live/dead cell staining. Each image was obtained from a confocal z-stack. **h** Mitotracker and Hoechst staining, obtained from confocal z-stacks

When MTX was administered for 6 days in 2D culture, cell viability differed between hepatocyte-like cells from healthy controls and hepatotoxic patients, but cell viability did not decrease below 50%. To decrease viability of MTX-treated cells below 50% and investigate the effect of long-term treatment with MTX, we tried to treat cells with MTX for more than 6 days. In the 2D-cultured state, apoptosis started to occur 30 days after hepatocyte differentiation. Hence, we decided to increase the survival period of hepatocytes by growing them in 3D spheroid culture. The hepatocyte spheroids were generated using the hanging drop system; we found that cell viability could be sustained for more than 40 days after differentiation when Matrigel matrix was present in the medium at a 1:100 ratio (Additional file 3). After hepatoblasts were mixed with basic Matrigel by centrifugation, the spheroids formed 3 days (healthy controls) or 7 days (RA patients) after aggregation (Fig. 4a, b). When hepatocyte spheroids were attached to the bottom of the dish after spheroid differentiation, the morphology of cells growing out from the spheroid was similar to that of primary hepatocytes [26] (Fig. 4a). IFA revealed that hepatocyte spheroids from both groups robustly expressed the hepatocyte markers albumin and A1AT (Fig. 4c). The fluorescence quantity per area of the spheroid is calculated and shown in a bar graph (Fig. 4d). As a result, the expression levels of albumin and A1AT were lower in RA hepatocyte-like cell spheroids compared to that of HC.

Starting on day 15 after hepatoblast aggregation, we administered MTX to 3D hepatocyte spheroids at a dose of 0 to 100 μM. Two weeks later, we performed ATP assays to measure cell viability. In the healthy control group, ATP level (RFU) increased up to a MTX concentration of 10 nM, and then gradually decreased from 1 to 100 μM. By contrast, in the RA patient group, the ATP level in the spheroids was higher after treatment with 10 nM MTX than after treatment with vehicle, and decreased significantly from 100 nM to 100 μM (Fig. 4e). Overall, the RA patient group had higher RFU values than the healthy control group. For determination of relative cell viability, the RFU value in vehicle was defined as 100% (Fig. 4f). Cell viability was below 50% at 1 μM MTX in the RA patient group, and at doses above 10 μM in the healthy patient group. Live/dead cell staining revealed that viability decreased relative to 0 nM MTX at doses above 100 nM (Fig. 4g).

To investigate drug-induced mitochondrial dysfunction, we stained with Mitotracker. The intensity of staining decreased in a MTX concentration-dependent manner in spheroids from the RA patient group (Fig. 4h), and the size of spheroids decreased at 10 and 100 μM MTX. The shapes of the RA patient spheroids were abnormal, and Hoechst staining revealed damage to the nucleus, at doses above 100 nM. No evidence of nuclear damage could be observed in the healthy control spheroids.

In summary, we successfully generated 3D hepatocyte spheroids from iPSCs that were suitable for long-term treatment with MTX. Hepatocyte spheroids derived from RA patients were more sensitive to 14-day MTX treatment than those obtained from healthy control cells. Moreover, mitochondrial and nuclear staining revealed abnormalities in the respective subcellular compartments in iPSC-derived hepatocyte spheroids from RA patients.

## Discussion

In this study, we generated 2D and 3D cultures of hepatocyte-like cells using iPSCs, and used these cultures to evaluate the hepatotoxicity of MTX. For cell differentiation, we used the Takebe protocol to differentiate iPSCs into hepatocyte-like cells via the endoderm and hepatoblast states [25]. In 2D culture, hepatocyte markers such as albumin, A1AT, and CK18 were expressed, and the differentiated hepatocyte-like cells had glycogen storage function and expressed drug metabolism genes such as phases I, II, and III. In addition, we could obtain hepatocyte-like cells from iPSCs derived from patients with hepatotoxicity following MTX administration, which could differentiate into albumin-positive hepatocyte-like cells at a yield of more than 80%. These cells could be used as a platform to test for drug toxicity. Remarkably, we were able to perform long-term treatment with MTX in iPSC-derived 3D hepatocyte spheroid cultures, whereas the short cellular lifespans limited the MTX treatment period to 6 days in 2D culture. In iPSC-derived 3D hepatocyte spheroids, cell viability was maintained for more than 40 days after the start of hepatoblast aggregation, allowing us to evaluate hepatotoxicity over long-term (14-day) treatment periods.

Recently, 3D culture methods for mimicking the in vivo environment, along with monolayer cultures, have been actively studied as a method for hepatocyte culture, with the ultimate goal of testing drug hepatotoxicity and verifying drug effectiveness. For example, in a 3D bioprinting system in which HepG2 hepatocarcinoma cells are cultured on an alginate scaffold, hepatocyte markers were robustly expressed, and scaffold-free microspheres of primary hepatocytes increased cellular lifespan and albumin secretion [31–34]. However, these studies were also limited by the difficulty of securing sufficient numbers of primary hepatocytes for drug testing. In addition, when iPSCs are differentiated into scaffold-free microspheres, the metabolic environment of hepatocytes is maintained more effectively in 3D culture than 2D culture. Patient characteristics about hepatotoxicity were not reflected in this case as well; however, our results verified that hepatocyte-like cells derived from iPSCs from healthy and RA patients with MTX-induced hepatotoxicity showed different sensitivity against drugs. In particular, it was possible to simulate the hepatotoxicity of long-term drug treatment by administering MTX to iPSC-derived 3D hepatocyte cultures for 14 days.

In this study, we generated hepatocyte-like cells from iPSCs to test the toxicity of MTX, which is already known to be hepatotoxic. In previous studies, iPSCs were generated from patients with hepatotoxicity due to pazopanib, for use as a disease model, and iPSCs derived from patients with A1AT mutations were differentiated into hepatocyte-like cells [35–38]. For differentiation into iPSCs, we used PBMCs or skin fibroblasts of RA patients, in which the levels of ALT and AST rose from 57 to 170 IU/L and 122 to 220 IU/L, respectively, after MTX administration. Mutant analyses of *MTHFR* [39], the candidate gene responsible for toxicity of MTX, revealed that the wild type, heterozygous mutants, and homozygous mutants were present in the patient group.

Pluripotent markers were expressed following iPSC differentiation: specifically, the genes encoding OCT3/4 and NANOG were expressed in cells derived from RA patients and healthy controls. iPSCs differentiated into hepatocyte-like cells that were more than 80% of which were albumin-positive (90.1% in healthy controls; 85.9% in RA patients) and expressed the hepatocyte markers A1AT and CK18. Therefore, iPSCs are a suitable source of material for evaluating a drug’s hepatotoxicity or efficacy. When cells were differentiated using the monolayer method, hepatocyte markers were expressed starting on day 18, so we administered MTX during maturation until day 26; this regimen was based on a consideration of lifespan limitations in 2D culture. Thus, 2D hepatocyte-like cells are a good model for verifying the hepatotoxicity and efficacy of drugs over a short period of time.

RA is a systemic autoimmune disease associated with bone destruction and inflammation. When used as a first-line drug, DMARDs run the risk of drug-induced liver injury, which in some patients is more dangerous than the disease. Several mechanisms have been proposed for MTX-induced liver injury, but the exact cause remains to be elucidated. Previous studies reported mutations in the DHFR and MTHFR enzymes [3, 5, 40]. Yet, it is not possible to predict hepatotoxicity using these mutations because they are not applicable in some cases. As for the revealed mechanism of MTX, MTX enters the cell by reduced folate carrier 1 reduce folate receptor (RFC-1; SLC19A1), is changed to methotrexate polyglutamates (MTXglu), and suppresses the intracellular 5-aminoimidazole4-carboxamide ribonucleotide (AICAR) transformylase. This leads to upregulation of intracellular AICAR, which leads to an increase in adenosine [29, 30]. Adenosine excreted in the extracellular fluid is known to bind to adenosine receptors A1, A2a with high affinity and A2b, and A3 with low affinity. In addition, ATP, ADP, and AMP are hydrolysis by CD39 and CD73 and increase adenosine. In particular, adenosine receptors A2a and A3 receptors are known to affect the anti-inflammatory effect and immunosuppression in the intracellular space [29, 30]. In a previous study, ABCC1, SLC19A1, ADORA2A, and ADORA2B were found to be upregulated in RA patient’ synovium that received MTX [29]. Another study has shown that the increase of adenosine by stimulation of A2B causes hepatic fibrosis [28]. In this study, ADOR A1, ADORA2B, CD39, and CD73 in iPSC-derived hepatocytes were expressed higher than HC in RA patient group with hepatotoxicity. In contrast, ADORA2A was expressed lower than HC in the RA patient group with hepatotoxicity. In addition, the expression of MDR1 (ABCB1) in the ATP binding cassette (ABC) transporter responsible for the release of MTX-Glu in the cells was more expressed in RA. As a result, the expression of ADORA1, ADORA2B, CD39, CD73 and MDR1 which are associated with adenosine binding was increased in RA hepatocyte-like cells, which might indicate more sensitivity to MTX. ADORA2A, which affects the anti-inflammatory effect, was decreased and ADORA2B, which may affect hepatic fibrosis, was increased in RA hepatocyte-like cells. These results suggest that the gene expression changes in RA hepatocyte-like cells may affect the outcomes related to hepatotoxicity.

In studies using Hep3B hepatocellular carcinoma cells, toxicity was observed at 100 nM MTX [41]. In another study using another HepG2, toxicity was 43.7% when treated with 100 μM MTX for 24 h and 17.69% when treated for 48 h [42]. Hepatocyte spheroids were prepared by mixing THP-1 and hTERT-HSC cells in HepaRG in a hanging drop system; cell viability was below 50% in 30 μM MTX after a 14-day treatment [43]. In other cases, primary hepatocyte was used to bio-print liver tissue with endotherial cells and hepatic stellate cells; when MTX was treated at 100 and 1 μM for 11 days and 14 days, LDH increased two to three times compared to vehicle and the results of the staining showed mild damage at 100 nM and fibrosis at 1 μΜ [44]. As far as we know, however, no previous study has examined MTX hepatotoxicity using patient-specific hepatocytes derived from iPSCs.

In this study, we generated hepatocyte-like cells using iPSCs, which can self-renew and provide patient-specific information. We found that the viability of hepatocyte-like cells from RA patients with hepatotoxicity was reduced when MTX was administered at doses from 100 nM to 100 μM for 6 days in 2D monolayer culture. When iPSC-derived 3D hepatocytes were treated with MTX for 14 days, viability of spheroids from RA patients decreased to 54.9% at 100 nM, 47.4% at 1 μM, and 16.7% at 100 μM. On the other hand, spheroids from healthy controls were comparatively resistant, with viability of 92.3% at 100 nM, 41.7% at 10 μM, and 23.5% at 100 μM. In the previous study, the toxicity of the carcinoma cell lines HepG2 and Hep3B was shown to be shorter in the treatment period of MTX than in the primary hepatocyte and iPSC-derived hepatocyte in this study. In contrast, the toxicity of MTX in liver tissue using primary hepatocytes, endothelial cells, and hepatic stellate cells in a previous study was similar to the period of MTX treatment and concentration with toxicity in this study. These results suggest that the toxicity study of this study system is similar to the toxicity seen in primary hepatocytes and reflects the sensitivity difference between healthy control and RA patients.

Mitochondrial dysfunction is a major mechanism underlying liver injury [45, 46]. In this study, the distribution of staining in RA patients with hepatotoxicity was abnormal, suggesting that RA patients with hepatotoxicity are more susceptible to mitochondrial dysfunction than healthy controls.

3D hepatocyte spheroids can be generated by the hanging drop method, in 96-well round-bottom plates, or by bioprinting [47]. In this study, cells were aggregated by centrifugation in 96-well round-bottom plates. Although iPSC-derived hepatocyte spheroids can be formed efficiently by the hanging drop method, we used a system in which one spheroid was present in each well, to allow treatment with a series of drug concentrations, and added Matrigel to the medium to form extracellular matrix and thereby maintain cell viability. Thus, the platform developed in this study can be used for a variety of assays of drug toxicity at a series of MTX concentrations.

## Conclusion

The toxicity of MTX was evaluated using iPSC-derived 2D hepatocyte-like cells and 3D hepatocyte spheroids. The results revealed that cultures derived from RA patients were more sensitive to MTX-induced hepatotoxicity than cultures derived from healthy control subjects. Because this system can be used to test hepatotoxicity for both short and long periods, it could be adapted into a test platform that could be monitored periodically. In addition, our findings provide evidence that patient-derived iPSCs could be used to study the mechanism of disease-related hepatotoxicity.

## Additional files

Additional file 1: Immunochemisty and AP staining of iPSCs. a, b Immunocytochemistry of HC- and RA-iPSCs. HC: healthy control; RA: RA patient with MTX-induced hepatotoxicity. c, d iPSCs generated from healthy controls and RA patients were positive for alkaline phosphatase staining. (JPG 21730 kb)

Additional file 2: Generation of iPSC-derived hepatocyte-like cells. a, b Morphology and immunocytochemistry images of iPSCs, endoderm, and hepatoblasts. On day 5, the endoderm marker SOX17 was expressed and pluripotency marker OCT3/4 was decreased. On day 8, the hepatoblast marker HNF4α was expressed and pluripotency marker OCT3/4 was decreased. Scale bars, 200 μm. (JPG 11851 kb)

Additional file 3: Generation of iPSC-derived hepatocyte spheroids using the hanging drop method, and survival period. a Scheme for generation of iPSC-derived hepatocyte spheroids. b Morphology of iPSC-derived hepatocyte spheroids during culture. Addition of Matrigel matrix (1:100 ratio in 25 μL of medium) increased spheroid survival rate. c Immunocytochemistry of iPSC-derived hepatocyte spheroids. Albumin and A1AT marker were expressed. Scale bars, 200 μm. (JPG 4520 kb)

## Abbreviations

A1AT: Alpha-1 antitrypsin
ADORA1: Adenosine A1 receptor
ADORA2A: Adenosine A2A receptor
ADORA2B: Adenosine A2B receptor
ADORA3: Adenosine A3 receptor
ALB: Albumin
bFGF: Basic fibroblast growth factor
BMP4: Bone morphogenetic protein 4
CK18: Cytokeratin 18
CYP3A4: Cytochrome P450 3A4
DMARDs: Disease-modifying antirheumatic drugs
HC: Healthy control
HGF: Hepatocyte growth factor
iPSC: Human-induced pluripotent stem cell
MTX: Methotrexate
NSAIDs: Nonsteroidal anti-inflammatory drugs
OSM: Oncostatin M
PBMC: Peripheral blood mononuclear cell
RA: Rheumatoid arthritis
