# Supplementary figures and images for: Recapitulation of methotrexate hepatotoxicity with induced pluripotent stem cell-derived hepatocytes from patients with rheumatoid arthritis
Source: Stem Cell Res Ther. 2018 Dec 29;9:357. doi: 10.1186/s13287-018-1100-1 (PMC6310944; doi:10.1186/s13287-018-1100-1)

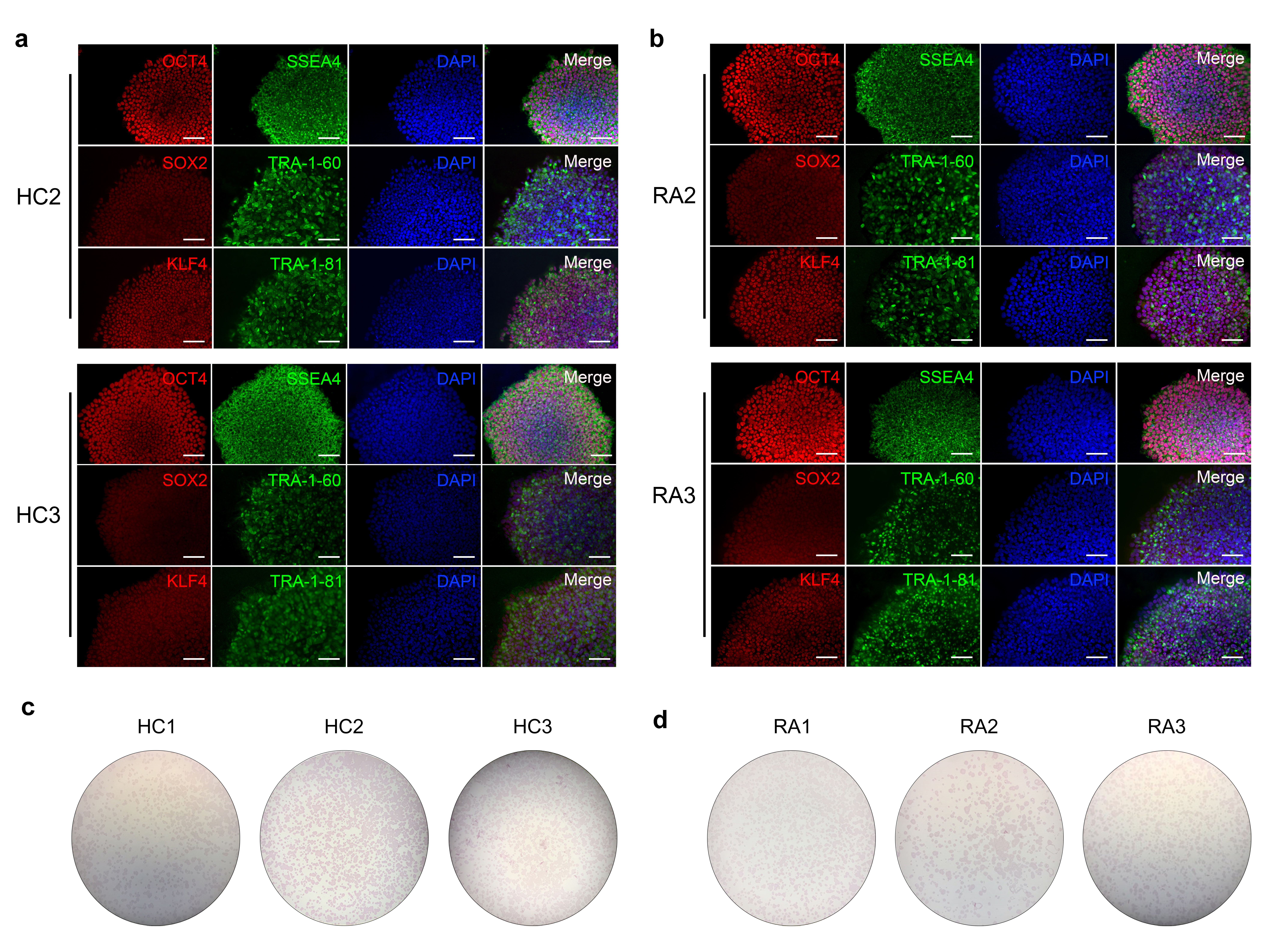

Supplement: Supplementary file 1 — Immunochemisty and AP staining of iPSCs. a, b Immunocytochemistry of HC- and RA-iPSCs. HC: healthy control; RA: RA patient with MTX-induced hepatotoxicity. c, d iPSCs generated from healthy controls and RA patients were positive for alkaline phosphatase staining. (JPG 21730 kb) [file 13287_2018_1100_MOESM1_ESM.jpg]

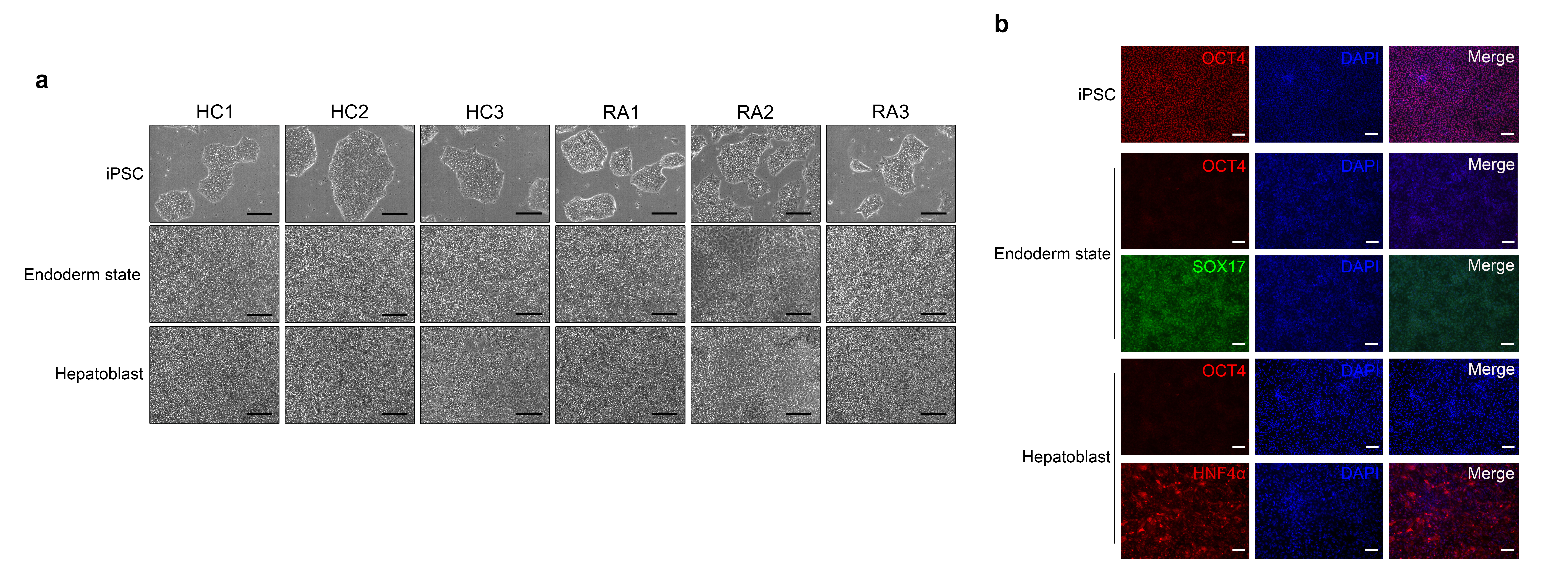

Supplement: Supplementary file 2 — Generation of iPSC-derived hepatocyte-like cells. a, b Morphology and immunocytochemistry images of iPSCs, endoderm, and hepatoblasts. On day 5, the endoderm marker SOX17 was expressed and pluripotency marker OCT3/4 was decreased. On day 8, the hepatoblast marker HNF4α was expressed and pluripotency marker OCT3/4 was decreased. Scale bars, 200 μm. (JPG 11851 kb) [file 13287_2018_1100_MOESM2_ESM.jpg]

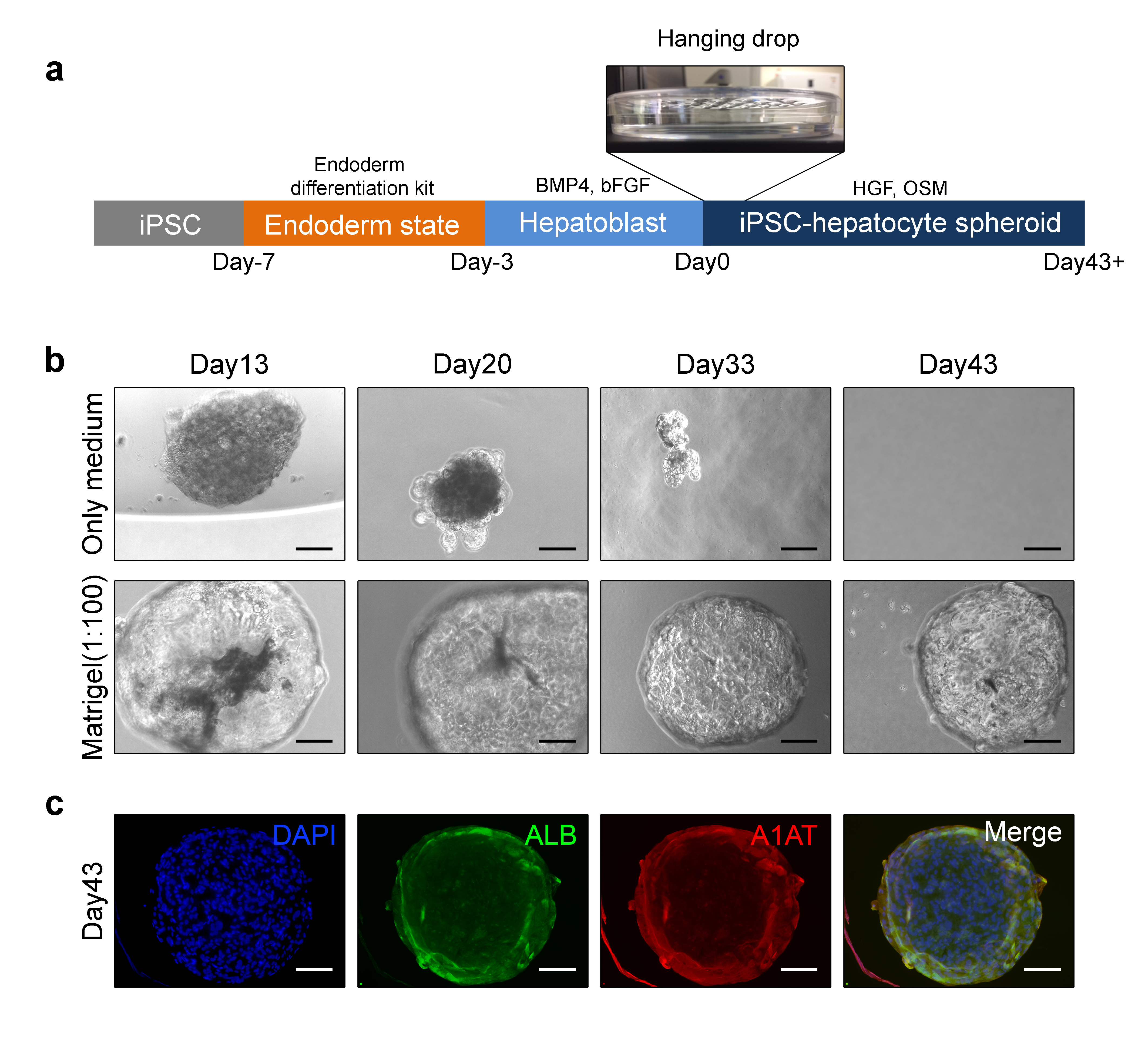

Supplement: Supplementary file 3 — Generation of iPSC-derived hepatocyte spheroids using the hanging drop method, and survival period. a Scheme for generation of iPSC-derived hepatocyte spheroids. b Morphology of iPSC-derived hepatocyte spheroids during culture. Addition of Matrigel matrix (1:100 ratio in 25 μL of medium) increased spheroid survival rate. c Immunocytochemistry of iPSC-derived hepatocyte spheroids. Albumin and A1AT marker were expressed. Scale bars, 200 μm. (JPG 4520 kb) [file 13287_2018_1100_MOESM3_ESM.jpg]
